# Accounting for size‐specific predation improves our ability to predict the strength of a trophic cascade

**DOI:** 10.1002/ece3.1870

**Published:** 2016-01-22

**Authors:** Christine F. Stevenson, Kyle W. Demes, Anne K. Salomon

**Affiliations:** ^1^ School of Resource and Environmental ManagementSimon Fraser University8888 University DriveBurnabyBritish ColumbiaCanadaV5A 1S6; ^2^ Hakai InstituteBritish ColumbiaCanada

**Keywords:** Biomass, body size, herbivory, size‐selective predation, trophic cascade

## Abstract

Predation can influence the magnitude of herbivory that grazers exert on primary producers by altering both grazer abundance and their per capita consumption rates via changes in behavior, density‐dependent effects, and size. Therefore, models based solely on changes in abundance may miss key components of grazing pressure. We estimated shifts in grazing pressure associated with changes in the abundance and per capita consumption rates of sea urchins triggered by size‐selective predation by sea otters (*Enhydra lutris*). Field surveys suggest that sea otters dramatically decreased the abundance and median size of sea urchins. Furthermore, laboratory experiments revealed that kelp consumption by sea urchins varied nonlinearly as a function of urchin size such that consumption rates increased to the 0.56 and 0.68 power of biomass for red and green urchins, respectively. This reveals that shifts in urchin size structure due to size‐selective predation by sea otters alter sea urchin per capita grazing rates. Comparison of two quantitative models estimating total consumptive capacity revealed that a model incorporating shifts in urchin abundance while neglecting urchin size structure overestimated grazing pressure compared to a model that incorporated size. Consequently, incorporating shifts in urchin size better predicted field estimates of kelp abundance compared to equivalent models based on urchin abundance alone. We provide strong evidence that incorporating size‐specific parameters increases our ability to describe and predict trophic interactions.

## Introduction

Mounting evidence suggests that variation in intraspecific traits such as body size can strongly influence the net effect of a species in a community (Bolnick et al. 2011). Body size is an important determinant of the direction and magnitude of species interactions and therefore can strongly influence the structure and dynamics of populations and communities. For instance, competition (Persson 1985), facilitation (Dickman 1988), food web stability (Emmerson and Raffaelli 2004), and choice of microhabitat (Dickman 1988) can shift with changes in body size. Additionally, the sizes of both predators and prey play a crucial role in predator–prey interactions (Lundvall et al. 1999; Toscano and Griffen 2011). Firstly, predators must be able to mechanically consume and process their prey and so predators are typically larger than their prey (Cohen et al. 2003). Likewise, some prey may escape predation by reaching an unmanageable size (Paine 1976; Brock 1979; Connell 1985). Secondly, prey capture efficiency and handling time are both related to the size of predators and prey (Evans 1976; Sousa 1993; Hirvonen and Ranta 1996).

Size‐selective predation contributes substantially to the ecological and evolutionary consequences of predator–prey interactions (Brooks and Dodson 1965; Sousa 1993; Bolnick et al. 2011). Size‐selective predators can impact prey directly through reducing their abundance (Paine 1976; Tonn et al. 1992) and by shifting the size structure of prey populations through selection of a preferred size (Kerfoot and Peterson 1980). The minimum prey size a predator is able to obtain depends upon a predator's aptitude of retaining (Persson 1987) or detecting (Lovrich and Sainte‐Marie 1997; Lundvall et al. 1999) prey, while the maximum prey size a predator selects is usually attributed to physical constraints (Hart and Hamrin 1988; Hambright 1991). Most theoretical explanations of prey size selection assume that predators consume prey that maximizes their net energy gain while foraging (Schoener 1969; Pyke 1984).

Grazers represent an important determinant of the structure and productivity of plant communities. Although a grazer's ability to consume plants is not usually physically constrained by plant size, the size of grazers can influence the grazing pressure exerted on plant communities by changing their per capita consumption rates of primary producers. Changes in per capita effects of grazers are important to consider because individual grazing rates may vary within a population. Many other factors can influence per capita grazing rates such as sex, developmental stage (Lundvall et al. 1999), physiological state, location, temperature (Cossins and Bowler 1987), predator cues (Garnick 1978; Scheibling and Hamm 1991), hydrodynamics (Kawamata 1997), density, and food availability (Holling 1959). However, body size will constrain consumption rate within all ecological and environmental contexts. A rich body of metabolic theory explains the relationship between body size and per capita consumption rates (Schmidt‐Nielsen 1984; Brown et al. 1993) as nonlinear, with small‐bodied organisms tending to have higher mass‐specific metabolic rates than larger‐bodied organisms. Because size‐selective predators consume grazers nonrandomly, models designed to estimate the indirect effects of predators on primary production should be more accurate if the changing size structure of the grazer population is considered in addition to changes in grazer abundance.

Predators play an important role in limiting grazers from depleting primary producers (Paine 1980; Carpenter et al. 1985; Shurin et al. 2002). Likewise, wide‐scale changes in predator dynamics, including either population depletion or recovery, can have profound indirect consequences for primary producers, as a result of altered grazing pressure (Estes et al. 2011). For example, eliminating the functional role of sea otters (*Enhydra lutris*) as apex predators from coastal temperate ecosystems has caused drastic shifts in nearshore rocky reef communities from kelp forests to urchin barrens, devoid of large macroalgae (Estes and Palmisano 1974). Sea otters are size‐selective predators that limit the abundance and size of their prey, especially sea urchins (Dayton 1975; Estes et al. 1978), which are important determinants in shaping nearshore benthic community structure because of their ability to excessively graze fleshy macroalgae (Duggins 1980; Harrold and Reed 1985; Byrnes et al. 2013). As otters move into a new rocky habitat, they preferentially select large and easily captured sea urchins first (Kvitek and Oliver 1988). We used this classic example of a tritrophic interaction to investigate the importance of changes in size structure as well as abundance of grazer populations in driving trophic cascades.

We used field survey data of sea urchin populations along a gradient of sea otter occupation time to investigate the influence of otter predation on the abundance and size structure of urchin populations. To test whether per capita kelp consumption rates of two urchin species varied as a function of body size, we measured kelp consumption rates across a variety of urchin sizes in controlled laboratory experiments. To assess whether nonrandom prey selection by otters changed the total grazing pressure of urchins on kelp, we compared two estimates of total consumptive capacity (TCC) across 13 sites occupied by sea otters: one based on urchin size structures unaffected by otter predation, and the other based on urchin size distributions resulting from the observed nonrandom prey selection by sea otters. TCC is defined as the theoretical maximum amount of kelp an urchin population could consume per hour assuming no resource‐dependent functional response. Finally, using survey data of urchin and kelp populations across 18 sites varying in sea otter occupation time from 0 to 33 years, we evaluated the relative strength of evidence of alternative candidate models of kelp abundance that differed in their proxy for grazing pressure.

## Methods

### Study area

**Figure 1:**
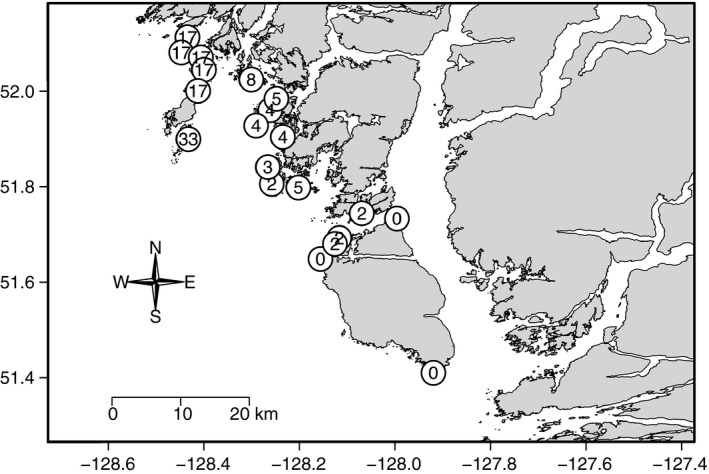
Site (*n* = 20) locations where surveys were conducted on the central coast of BC, Canada. Numbers represent otter occupation time (in years) of each site.

Following the extirpation of sea otters from the northeast Pacific, recovering populations of this keystone predator have been expanding their range (Larson et al. 2015). On the central coast of British Columbia (BC), Canada sea otter observations were first reported in 1989 and the population has since increased at a rate of 11% a year and expanded its range both southwards and northwards (Nichol et al. 2009). We took advantage of the expanding margins of the sea otter population in this region and sampled urchin populations at 20 rocky reefs situated along approximately 150 kms of coastline following a gradient in sea otter occupation time (0–33 years; Fig. 1). Sites were considered “occupied” following the sighting of a raft (≥3 individuals) within a three nautical miles radius of each site based on boat surveys and confirmed raft sightings (Nichol et al. 2009). The occupation time of each site was established based on extensive sea otter surveys conducted every 1–3 years by Fisheries and Oceans Canada beginning in 1990 and most recently in 2013 (Nichol et al. 2015; for full details on survey methods see Nichol et al. 2009). Red urchins (*Mesocentrotus franciscanus*), green urchins (*Strongylocentrotus droebachiensis*), and purple urchins (*Strongylocentrotus purpuratus*) are all present in the study area. We focused on red and green urchins because they accounted 99.6% of all urchins sampled.

### Site selection

We chose our sites by identifying subtidal rocky reefs that had similar physical characteristics (i.e., depth range, wave exposure, aspect, and topography) but varied in sea otter occupation time, from 0 to 33 years. We used reconnaissance dives to ensure that each reef encompassed at least 100 m of continuous rocky substrate.

### Sea otter occupation time

Unlike most studies of sea otter‐induced trophic cascades, we used sea otter occupation time rather than sea otter density to estimate variation in predation pressure across our 20 sites. We focused on the magnitude of sea otter predation pressure as a function of time due to the natural history of sea otter range expansion and occupation. Sea otter rafts are typically segregated by sex (Riedman and Estes 1990), with male rafts tending to occupy the periphery of the population range. Range expansion occurs in growing populations when large rafts of males appear in previously unoccupied urchin barren habitat (Garshelis et al. 1984) followed later by smaller rafts of females with pups once male rafts have left. Thus, high sea otter densities often occur at newly occupied urchin barren sites, while lower densities occupy recovered forested sites. Consequently, the ecological condition that drives the transformation between urchin barrens and kelp forests is the sustained press perturbation of predation pressure measured over time rather than sea otter density measured at a particular moment in time. While an ideal measure of sea otter predation on sea urchins would be a composite variable of both the number of otters and the duration of their occupation at a site, high‐resolution spatial and temporal abundance data do not exist for this remote region.

### Assumptions of space‐for‐time substitutions

While valuable in the absence of time series data, space‐for‐time substitutions have their limitations (Pickett 1989; Vitousek et al. 1997). Importantly, they assume that the variable of interest, in this case sea otter occupation time, is imposed randomly on the landscape and that sites differ only due to this variable. In reality, variables other than sea otter occupation time such as currents, wave energy, water temperature, and sea urchin recruitment rates likely differed among our sites and may have also affected sea urchin size structure across our sites. Nonetheless, we provide strong evidence that sea otter occupation time is the dominant driver of urchin size structure across our sites.

### Field surveys

#### Urchin abundance and size across the predator gradient

Sea urchins were counted and measured in 1 m^2^ quadrats (*n* = 18) randomly stratified between 4 and 15 m below chart datum at each site. Urchin size was recorded as the maximum test diameter to the nearest centimeter. Red urchins were observed in quadrats at 18 of our 20 sites, while green urchins were only recorded at 12 sites. In each quadrat, kelp density was also determined by counting the number of stipes (>15 cm).

### Laboratory experiment

#### Urchin size‐specific grazing rates

To determine the size‐specific consumption rates of red and green urchins, grazing trials were conducted in flow‐through seawater tanks. Red and green urchins were collected by divers in June 2014 from Barkley Sound and transported in coolers directly to the laboratory. An effort was made to collect organisms from the entire size range in order to represent the full size distribution of each population, resulting in 30 red urchins (13–155 mm) and 36 green urchins (15–64 mm). Maximum test diameter was measured with digital calipers to the nearest mm. Giant kelp, *Macrocystis pyrifera*, was collected and used in the feeding trials because it commonly occurs in urchin habitat, is heavily grazed by both species of urchin (Tegner and Dayton 1981, Wilson et al. 1977), is the preferred macroalga of both urchin species (Leighton 1966, North 1971), and has a high digestibility compared to other kelp species (Vadas 1977).

Urchins were placed in enclosed grazing arenas consisting of inverted plastic containers with holes in the sides to allow for seawater circulation. In order to optimize tank space while still ensuring that each urchin had ample space to move, plastic containers varied in size based on the size of each organism. A single urchin was placed in each container with the exception of one container used as a control to assess any potential loss or gains in *M. pyrifera* during experiments due to factors other than grazing (i.e., erosion or growth). After approximately 48 h without feeding, fresh *M. pyrifera* (7.3 ± 0.1 g) was added once to every container at the beginning of each trial. Because of space constraints we ran red and green urchin feeding trials separately for each species, but all grazing trials were run simultaneously. Grazing rates were calculated based on the amount of kelp (g) consumed per hour; therefore, to avoid underestimating grazing rate due to the complete consumption of kelp, we ensured that at least 30% of each kelp sample remained at the end of every trial. Accordingly, green urchins were allowed to graze for 7 h and red urchins for 5 h as red urchins consumed the kelp more quickly. Red urchins grazed faster and more variably than green urchins, so we repeated grazing trials on the same red urchins three times and the mean grazing rate of each urchin (*n* = 30) was used in the final analysis.

Photographs were taken of each *M. pyrifera* sample before and after each trial and image analysis was used to calculate kelp consumed as the change in surface area. A surface area‐to‐wet weight linear regression was used to convert surface area of kelp from photographs into grams (kelp_consumed (grams)_ = 0.04 × area_lost(cm_^2^_)_, *P* < 0.001, *R* ^2^ = 0.96, *n* = 50). We accounted for the image software calibration error across all samples.

### Data analysis

#### Model selection

We determined the relative strength of evidence among alternative candidate models of our empirical data using an information‐theoretic approach (Burnham and Anderson 2002). We used small‐sample bias‐corrected Akaike's information criterion (AIC_c_) to rank each candidate model, standardized to the most parsimonious model to produce ∆AIC_c_ values (Burnham and Anderson 2002). AIC_c_ considers goodness of fit but also includes a penalty that increases with the number of estimated parameters; therefore, the model with the lowest AIC_c_ value is best. We normalized the model likelihoods to a set of positive Akaike weights (*W*_*i*_) representing the relative strength of evidence for each model. The nonlinear least squares (nls) function in r statistical software was used to fit alternative candidate models to all of the regression data.

#### Urchin abundance and size across the predator gradient

Linear and exponential models were chosen to evaluate the effect of otter occupation time on urchin abundance and size in order to assess whether sea otters consume urchins continuously or if consumption slows down as urchin populations start to decline (e.g., decreased capture success for sparse populations, diminished energetic incentive to eat smaller individuals). An intercept model was used as a null model describing the absence of an effect of otter occupation time on urchin abundance or size. To describe the relationship between sea otter occupation time and abundance of each urchin species, we determined the mean urchin density (urchins/m^2^) at the site level and evaluated the relative strength of evidence between three alternative models of urchin density as a function of otter occupation time: an intercept‐only null model, a linear model, and an exponential model. To visually examine changes in urchin size structure resulting from size‐selective predation, we constructed size frequency distributions of red and green urchins separated into 3 categories of otter occupation time based on clear breakpoints in urchin density presented in Fig. 1(A) and representing similar sampling effort (*n* = 6–7 sites): low (0–2 years), intermediate (3–8 years), and high (17–33 years) otter occupation time. For each urchin species, the median size was determined and compared for each otter occupation category to illustrate change in median size with increasing predation pressure. To better describe the effect of otter occupation time on urchin size, we determined median size of each species at the site level and determined the relative strength of evidence of three alternative models of median urchin size as a function of otter occupation time: an intercept‐only null model, a linear model, and an exponential model.

#### Urchin size‐specific grazing rates

To parameterize the relationship between individual urchin biomass and its kelp consumption rate, we compared the relative strength of evidence of a power model to an intercept‐only null model. We fit a power model to the urchin grazing data since a power model is commonly accepted for animal metabolic rates (Fuji 1962; Miller and Mann 1973; Hamburger et al. 1983; Kawamata 1997; Fidhiany and Winckler 1998). Red urchin biomass was estimated from test diameter‐to‐weight regression analysis based on roughly 10,000 red urchin samples from Tofino, Price Island, and Haida Gwaii, BC (Fisheries and Oceans Canada database 2015). Green urchin biomass was estimated from test diameter‐to‐weight regression analysis using the test diameters and weights of the 40 individuals from the grazing experiment.

#### Calculation of total consumptive capacity

To calculate total consumptive capacity of urchin populations in the field, we first used the same test diameter‐to‐weight regressions as described in the grazing trials to convert the test diameter of each red and green urchin to biomass. We then estimated how much kelp each red and green urchin in the field could graze per hour using the regression equations parameterized in the grazing trials. Total consumptive capacity (TCC) of an urchin population was then calculated by summing the estimated kelp consumption of all urchins in an area (i.e., site vs. quadrat depending on analysis). Red and green urchin TCCs were combined in order to assess the collective effect of urchin populations on kelp across sites.

#### Impacts of urchin size on total consumptive capacity and kelp abundance

To test whether otter‐induced shifts in urchin size structure change the consumptive pressure of urchins, we used a paired *t*‐test to compare TCC at the site level estimated under two scenarios: (1) Only urchin abundance is affected by otter predation (i.e., urchin sizes were randomly drawn from the size structure of urchins in sites with no otter predation), and (2) otter predation affects both urchin abundance and size structure (i.e., the actual observed size of each urchin in sites affected by otters). In order to reflect an urchin abundance affected by otter predation, only the sites with otters present (*n* = 13) were used for comparison. We then tested whether incorporating changes in urchin size structure better predicted shifts in kelp abundance than urchin abundance alone by comparing the fit of alternative candidate models of kelp abundance based on urchin density, biomass, and TCC at the 1 m^2^ quadrat level (*n* = 358). We also considered the commonly accepted scaling relationship between biomass and metabolic biomass (mass^0.75^) compared to density, biomass, and TCC as a predictor of kelp abundance because previous studies have found a similar relationship between urchin mass and kelp consumption (Miller and Mann 1973). We determined the relative strength of evidence for an intercept‐only null model and a linear and exponential model for each of these four predictor variables (urchin density, summed urchin biomass, TCC, and summed metabolic biomass). Analysis was done by quadrat rather than by site because urchins have a quadrat level effect on kelp abundance (Salomon and Demes 2015).

## Results

### Urchin abundance and size across the predator gradient

**Figure 2:**
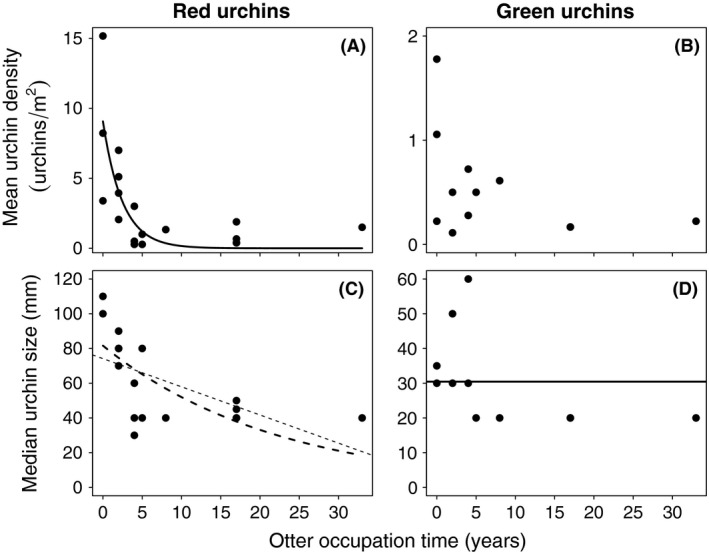
Change in (A) red and (B) green urchin density and (C&D) size with increasing otter occupation time. The solid lines represent parsimonious models with greatest strength of evidence. The dotted lines represent alternative candidate models, as there was not strong support for one over the other. No line represents that there was not strong support for the alternative models over the intercept (null) model. Note differences in *y*‐axis scale among graphs.

**Figure 3:**
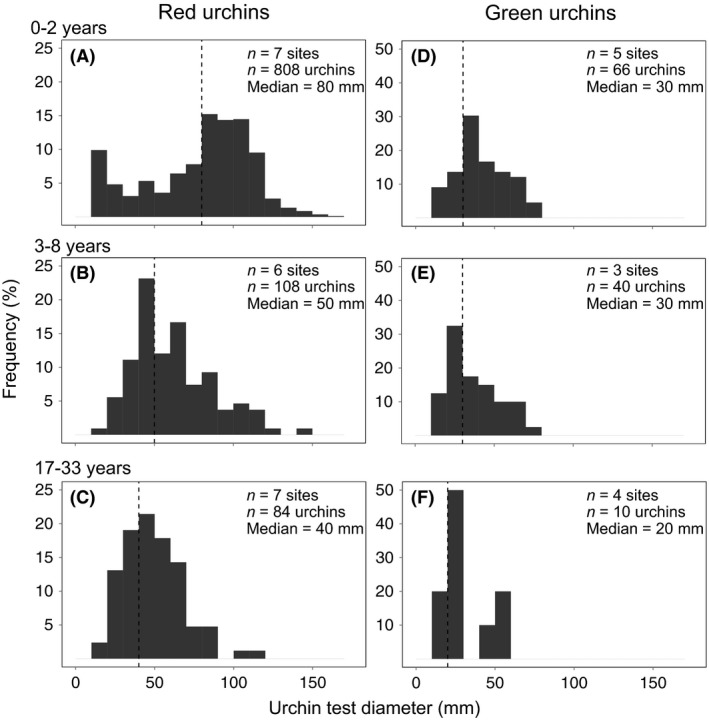
Red (A–C) and green (D–F) urchin test diameter size frequency distribution separated by low (0–2 years), intermediate (3–8 years), and high (17–33 years) categories of otter occupation time. Dotted line represents median size of red and green urchins within each sea otter occupation time category and is noted in graph legend. Bin size = 10. Note differences in *y*‐axis scale among graph panels.

Red urchin density decreased with otter occupation time (Fig. 2A) and this relationship was best described as an exponential decline (*w*_*i*_ = 0.99, *R* ^2^ = 0.59, Table 1). On the other hand, we did not find evidence of an association between otter occupation time and green urchin density (Fig. 2C), as the linear and exponential models were indistinguishable from the intercept null model (Table 1).The red urchin size frequency distribution for low otter occupation sites revealed a bimodal population size structure (Fig. 3A), while intermediate and high otter occupation sites were unimodal, lacking large (>80 mm) and juvenile (<20 mm) urchins (Fig. 3B and Fig. 3C). The median red urchin size for low, intermediate, and high otter occupation categories was 80 mm, 50 mm, and 40 mm, respectively. Site‐level analyses revealed decreasing red urchin median size with increasing otter occupation time (Fig. 2B) with comparable support in the data for a linear and an exponential model (Table 1). The green urchin size frequency distribution showed a less distinct absence of larger individuals with increasing predation pressure. The median urchin size for low, intermediate, and high otter occupation was 30 mm, 30 mm, and 20 mm, respectively. However, since the intercept null model was the top model, there was no evidence of an effect of otter occupation time on green urchin median size (Fig. 2D, Table 1).

**Table 1:** Strength of evidence for alternative candidate models of the spatial variation in red and green sea urchin mean density and median size across sites varying in sea otter occupation time. Models with varying numbers of parameters (*K*) were compared using differences in small‐sample bias‐corrected Akaike information criterion (∆AIC_c_), and normalized Akaike weights (*W*_*i*_). Bold typeface indicates a model that has substantial empirical support relative to alternative candidate models

Response and Model	*n*	*K*	AIC_c_	ΔAIC_c_	rlikelihood	*W*_*i*_	Pseudo *R* ^2^	Parameters
Mean red urchin density								
**Exponential**	**18**	**2**	**90.06**	**0**	**1.000**	**0.991**	**0.59**	***a*** ** = −9.01; ** ***b*** ** = 0.684**
Linear	18	2	102.02	12	0.003	0.003	0.15	*b* = 4.61; *m* = **−**0.191
Intercept	18	1	103.09	13	0.001	0.001	NA	Mean = 3.12
Mean green urchin density								
Exponential	12	2	20.73	0	1.000	0.396	0.21	*a* = **−**0.859; *b* = 0.887
Intercept	12	1	20.90	0.2	0.919	0.364	NA	Mean = 0.528
Linear	12	2	22.07	1.3	0.512	0.203	0.12	*b* = 0.689; *m* = **−**0.021
Median red urchin size								
Exponential	18	2	164.58	0	1.000	0.503	0.41	*a* = 76.6; *b* = 0.963
Linear	18	2	166.26	1.7	0.432	0.217	0.30	*b* = 71.7; *m* = 1.49
Intercept	18	1	169.68	5.1	0.078	0.039	NA	Mean = 60.3
Median green urchin size								
**Intercept**	**12**	**1**	**101.96**	**0**	**1.000**	**0.588**	NA	**mean = 28.75**
Exponential	12	2	104.13	2.2	0.337	0.198	0.19	*a* = **−**32.86; *b* = 0.9807
Linear	12	2	104.16	2.2	0.332	0.195	0.16	*b* = 32.44; *m* = **−**0.4814

### Urchin size‐specific grazing rates

**Figure 4:**
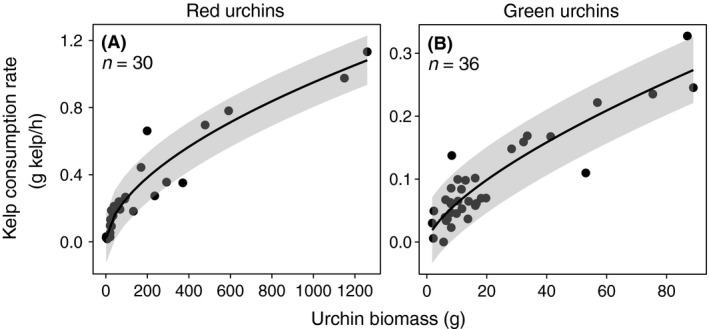
(A) Red urchin and (B) green urchin size‐specific grazing rates. (Grazing_Red_ = 0.02 × Biomass_Red_^0.56^). (Grazing_Green_ = 0.01 × Biomass_Green_^0.68^). Solid line represents power functions fit to the data. Gray area represents 95% confidence intervals.

Urchin test diameter (mm) was a strong predictor of biomass (*g*) for both species: Biomass_Red_ =0.000969 × Diameter_Red_^2.79^ (*R* ^2^ = 1.00) and Biomass_Green_ =0.001124 × Diameter_Green_^2.71^ (*R* ^2 ^= 0.99). In both red and green urchins, per capita grazing rate (g kelp/h) increased rapidly for smaller individuals but began to saturate with increasing individual urchin biomass (Fig. 4A and B). The power models fit to the relationship between grazing rate and urchin biomass for red and green urchins were the following: Grazing_Red_ = 0.02 ×Biomass_Red_^0.56^ (*w*_*i*_ = 1.0, *R* ^2^ = 0.91) and Grazing_Green_ =0.01 × Biomass_Green_^0.68^ (*w*_*i*_ = 1.0, *R* ^2^ = 0.82). For both species, there was no relative evidence for an intercept null model (*w*_*i*_ <0.001).

### Impacts of urchin size on total consumptive capacity and kelp abundance

**Figure 5:**
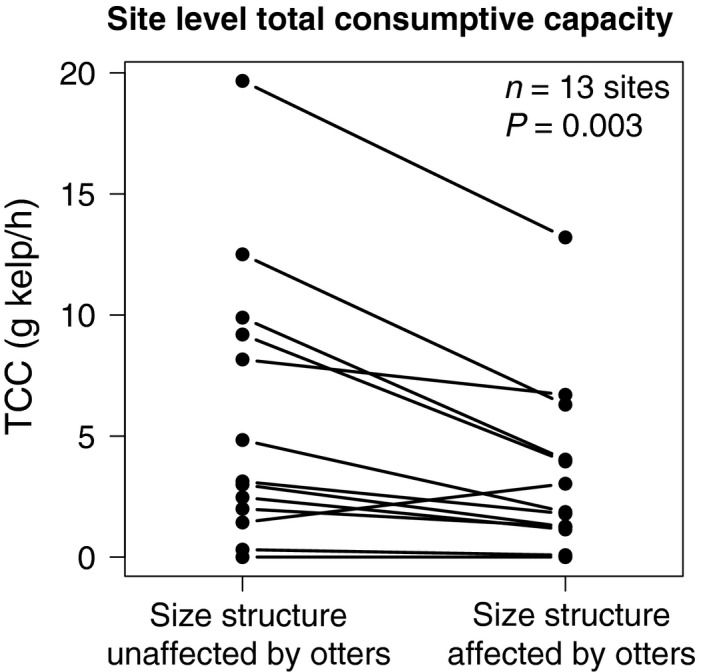
Estimated total consumptive capacity (TCC) for urchin populations with no impact on size distributions versus estimated total consumptive capacity for urchin populations resulting from size‐selective predation. Only sites with otters present were compared (*n* = 13).

**Figure 6:**
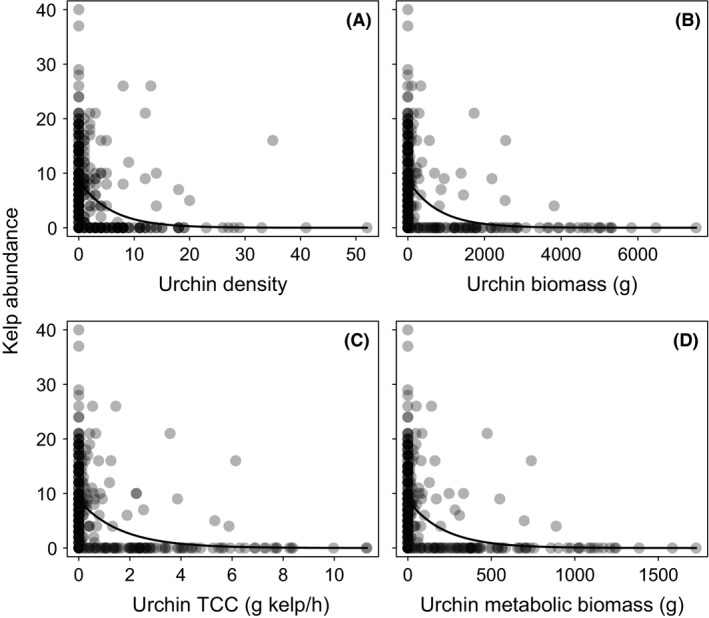
Comparative models of kelp density as a function of (A) urchin density (B) urchin biomass, (C) urchin TCC, and (D) urchin metabolic biomass. Solid line represents the exponential models fit to each set of data. Darker shading of data points indicates higher frequency of overlapping points. *N* = 358 1 m × 1 m quadrats.

Total consumptive capacity of urchins at each site was significantly lower (*P* = 0.003) when incorporating the changes in size structure than when using grazing rates corresponding to randomly assigned sizes to each urchin, although the magnitude of difference varied across sites (Fig. 5). Kelp abundance at the quadrat level decreased rapidly with increasing urchin density, biomass, total consumptive capacity, and metabolic biomass (Fig. 6). The exponential model incorporating shifts in urchin biomass resulting from shifts in size structure of urchin populations had the strongest and most parsimonious explanatory power to predict kelp abundance (*w*_*i*_ = 0.97, Table 2).

**Table 2:** Strength of evidence for alternative candidate models of kelp stipe density as a function of urchin density, biomass, TCC, and metabolic biomass. Models with varying numbers of parameters (*K*) were compared using differences in small‐sample bias‐corrected Akaike information criterion (∆AICc), and normalized Akaike weights (*Wi*). All models were compared simultaneously. Bold typeface indicates a model that has substantial empirical support relative to all alternative candidate models

Predictor and Model	*n*	*K*	AIC_c_	ΔAIC_c_	rlikelihood	*W*_*i*_	Pseudo *R* ^2^	Parameters
Urchin Biomass								
**Exponential**	**358**	**2**	**2354.09**	**0**	**1**	**0.972**	**0.18**	***a*** ** = −8.76; ** ***b*** = **0.999**
Linear	358	2	2375.18	21	<0.001	<0.001	0.13	*b* = 8.02; *m* = **−**0.002
Intercept	358	1	2425.08	71	<0.001	<0.001	NA	mean = 6.81
Urchin Metabolic Biomass								
Exponential	358	2	2357.49	3	0.182	0.177	0.17	*a* = **−**8.76; *b* = 0.995
Linear	358	2	2376.36	22	<0.001	<0.001	0.13	*b* = 8.05; *m* = 0.009
Intercept	358	1	2425.08	71	<0.001	<0.001	NA	mean = 6.81
Urchin TCC								
Exponential	358	2	2361.17	7	0.030	0.028	0.17	*a* = **−**8.76; *b* = 0.546
Linear	358	2	2378.87	25	<0.001	<0.001	0.12	*b* = 8.05; *m* = **−**0.389
Intercept	358	1	2425.08	71	<0.001	<0.001	NA	mean = 6.81
Urchin Density								
Exponential	358	2	2379.17	25	<0.001	<0.001	0.13	*a* = **−**8.72; *b* = 0.843
Linear	358	2	2399.34	45	<0.001	<0.001	0.07	*b* = 7.76; *m* = **−**0.300
Intercept	358	1	2425.08	71	<0.001	<0.001	NA	mean = 6.81

## Discussion

Size‐selective predators strongly influence community dynamics by changing the abundance and size structure of grazer populations (Dayton 1971; Sprules 1972; Paine 1976). While the role of size‐selective predation in driving the magnitude of trophic cascades has been revealed in lake systems (e.g., Schindler et al. 1993; Twining and Post 2013), it has yet to be investigated in the context of the cascading effects triggered by a marine mammal. Here, we show that size‐selective predation by sea otters dramatically changes the abundance and size structure of red sea urchin populations. Red urchins were markedly reduced in numbers with increasing otter occupation time and those remaining were small individuals likely because small red urchins may not be worth the trade‐off between the energy expended for collection and the energy gained by consumption (Schoener 1969; Pyke 1984). Additionally, otters may have a limited ability to detect (Lovrich and Sainte‐Marie 1997; Lundvall et al. 1999) or retain (Persson 1987) small red urchins as prey. The median size of red urchins dropped by 63%, a result of the removal of the largest individuals shortly after otter arrival. We found strong evidence for a negative effect of otter predation on median red urchin size, but our dataset did not distinguish which alternative model (e.g., linear vs. nonlinear) best described this effect (Fig. 2C). Green urchins on the other hand did not experience a change in abundance or size as a function of otter occupation time. This lack of effect is likely because green urchins may not be as energetically beneficial or as easy for otters to detect or retain as red urchins due to green urchins' relative rarity and comparatively smaller size. Green urchins only accounted for about 11% of all urchins sampled, their maximum test diameter was 70 mm, whereas the maximum red urchin test diameter was 160 mm, and most green urchins were smaller overall than most of the red urchins remaining in areas with high otter occupation time.

Our results show that size‐selective predation on grazers can have cascading effects on plant populations beyond those predicted by shifts in grazer abundance alone. Predators can shift per capita consumptive capacity of grazers by altering grazer density, behavior, and size of grazers. Although density dependence (Holling 1959) and behavioral (Watson and Estes 2011) traits can influence per capita grazing rates, TCC is the theoretical maximum amount of kelp urchins could consume since it is based on ad libitum grazing trials in the absence of predators. Because we did not empirically consider density dependence and behavioral shifts, we cannot address those factors here. Although many other factors can influence per capita grazing rate of urchins (e.g., De Ridder and Lawrence 1982; Lawrence 1987; Lawrence et al. 2006), body size will ultimately constrain grazing rates (Hillebrand et al. 2009). Our data reconfirm and parameterize size‐dependent grazing rates of red and green urchins (Fig. 4A and 4B) and show that shifts in urchin size from size‐selective predation by otters have important implications for the grazing potential of urchins on kelp in the absence of behavioral shifts or density dependence. By specifically quantifying the size‐specific grazing rates, we were able to measure a mechanism responsible for the impact on primary production and better understand the role that predators and grazers play within ecosystems.

Metabolic theory and a rich body of empirical evidence (e.g., Kunetzov 1946; Fuji 1962; Moore and McPherson 1965; Peters and Downing 1984; Carpenter et al. 1985; Kawamata 1997) highlight that absolute consumption rates increase, whereas mass‐specific rates decrease with animal biomass. The power curves fit to our grazing data are consistent with this trend and demonstrate that larger urchins eat more kelp than smaller urchins. This is expected as the size of an urchin's Aristotle's lantern can be directly related to their feeding rate on algae (Black et al. 1984). Metabolic theory also states that small organisms have a higher metabolic rate per unit of weight than large ones of the same species (Hemmingsen 1960). In most groups of organisms, the exponent for the biomass relation to metabolic rate is commonly accepted to be about 0.75 (Schmidt‐Nielsen 1984). For urchins, Miller and Mann (1973) found that consumption rate (cal/urchin/day) calculated from direct measurements of the calorific value of kelp consumed per urchin is proportional to the 0.73–0.87 power of animal biomass (g). Our model predicts that grazing rate (g kelp/h) measured by the total amount of kelp consumed by each urchin is proportional to the 0.56 and 0.68 power of red and green urchin biomass (g), respectively. The difference in the use of the calorific value of kelp consumed and the total grams of kelp consumed as measures of consumption rate may account for the difference in exponents.

The nonlinear relationship between individual urchin biomass and grazing rate described by power curves with exponents <1 highlights the importance of incorporating size‐specific grazing rates because it suggests that even using biomass instead of abundance of grazers would not be expected to accurately predict grazing pressure, as the two are nonlinearly related. However, our study suggests that biomass may account for the total consumptive capacity of grazers because although grazing rate may be a more direct measure of grazing pressure, the model using biomass was a better predictor of kelp abundance data than the model using TCC (Table 2). This may be due to propagated error associated with estimating TCC from test diameter: There are first uncertainties associated with estimating biomass from test diameter and then there is also error when estimating TCC from biomass using our regression model. Furthermore, TCC may not predict plant dynamics better than biomass if all grazers in the population fall within a narrow size range, either on the first or second portion of the curves represented in Fig. 3(A&B), for which increases in size may result in approximately linear increases in grazing rate. However, further examination of the relationship between red urchin biomass and grazing rate for red urchins within the 90th percentile of biomass revealed that the power model maintained its explanatory power compared to a linear model (Linear: AIC_c_ = 21.82, *w*_i_ = 0.04; Power: AIC_c_ = 15.20, *w*_i_ = 0.96). Since red urchins accounted for 89% of all urchins sampled in the field, the cumulative error associated with estimated TCC is more likely the reason urchin biomass better predicts kelp abundance than TCC.

Previous studies suggest that the impact of a grazer population on plant communities largely reflects the metabolic demand and constraints of grazers and thus metabolic biomass may be a reliable predictor of grazing pressure (Chalcraft and Resetarits 2004; Schmitz and Price 2011; Atkins et al. 2015). Our results suggest that metabolic biomass is a better predictor of plant abundance than total consumptive capacity of grazers, which may be due to less error amplification associated with metabolic biomass than TCC. Because TCC was determined under laboratory conditions and over a relatively short time period, it may not translate well into consumption rates exhibited in the field. The theoretical prediction of metabolic biomass may better reflect conditions experienced in the field and over longer periods of time. However, our results demonstrate that although the model of metabolic biomass was more parsimonious than TCC, urchin biomass remained the best predictor of kelp abundance. While size‐specific grazing rates or metabolic biomass may not be necessary to evaluate total potential grazing pressure, grazer size remains important to consider because the model including biomass to describe kelp abundance was considerably more parsimonious than the model consisting of density alone. Although biomass was the best predictor of kelp abundance compared to our other variables, its low explanatory power (*R* ^2^ = 0.18) highlights that urchin biomass is only one of many important elements influencing kelp abundance across sites.

Sea otters are not the sole driver of urchin population dynamics and a number of additional factors may also influence urchin size structure (e.g., currents, wave exposure, water temperature, urchin harvest, additional predators such as *Pycnopodia*, and variable urchin recruitment rates). However, sea otters are widely recognized as aggressive size‐selective predators that alter sea urchin populations dramatically across their distribution, including in our study region (Honka 2014). Sea otters on the central coast of BC can consume up to 50 urchins per hour after recently colonizing a site and urchins consist of almost 90% of their diet (Honka 2014). Honka (2014) found that after only 1 year of occupation, sea otter per capita urchin consumption rates dropped by 70% due to decreased urchin density following intense predation in the first year. Furthermore, sea otters are known to specifically select larger urchins first when moving into new sites (Honka 2014). Likewise, we posit that the influence of other extraneous factors on sea urchins in our system is overwhelmed by the dramatic foraging behavior of sea otters.

Many studies have focused on how changes in grazer abundance are an important factor in determining abundance of primary producers (Breen and Mann 1976; Mann 1977; Milchunas et al. 1988). However, because intraspecific trait variation among a population can have significant ecosystem effects, overlooking grazer size leads to an incomplete understanding of grazing pressure. Although urchin abundance is certainly important in grazer–producer interactions, results of this study suggest that incorporating size significantly increases our ability to predict patterns of plant abundance. The distinction between abundance and size is important given that metabolic rates depend on body size and determine the rate of food consumption (Chalcraft and Resetarits 2004; Schmitz and Price 2011). Recent work on salt marsh plants demonstrated that when the mean body size of snails is shifted, metabolic biomass (mass^0.75^) is the best predictor of grazing damage to primary producers when competed against biomass or density models (Atkins et al. 2015). Our study demonstrates that such an assumption may not maintain its explanatory power when grazer body size is shifted across the entire grazer population size range. Rather, here biomass better predicts impact on primary producers than metabolic biomass and grazing rate.

Size‐specific parameters can be easily incorporated into ecological models and enhance our ability to describe species interactions and predict trophic cascades by accounting for a common and large source of variation in per capita interaction strength: size. Our study shows that biomass is a more accurate descriptor of the total potential effect of grazers on plant communities and emphasizes the importance of including biomass to increase the predictive power of ecological models.

## Conflict of Interest

None declared.
